# Supplementary material for: Predicting Growth and Carcass Traits in Swine Using Microbiome Data and Machine Learning Algorithms
Source: Sci Rep. 2019 Apr 25;9:6574. doi: 10.1038/s41598-019-43031-x (PMC6484031; doi:10.1038/s41598-019-43031-x)
Supplement: Supplementary file 1 — Supplementary Material [file 41598_2019_43031_MOESM1_ESM.docx]

**Predicting Growth and Carcass Traits in Swine Using Microbiome Data and Machine Learning Algorithms**

Christian Maltecca^1,*,+^, Duc Lu^1,+^, Constantino Schillebeeckx^2^, Nathan P McNulty^2^, Clint Schwab^3^, Caleb Shull^3^, and Francesco Tiezzi^1,+,*^

1North Carolina State University, Animal Science Department, Raleigh, 27695, USA

2Matatu Inc., Saint Louis, 63108, USA

3The Maschhoffs LLC, Carlyle, 62231, USA

*Correspondence and requests for materials should be addressed to C.M. (cmaltec@ncsu.edu), or F.T. (f tiezzi@ncsu.edu)

+These authors contributed equally to this work

| **Estimate** | **SE** | **p-value** | **FDR** | **Trait** | **Age** | **Name** |
| --- | --- | --- | --- | --- | --- | --- |
| -0.00576 | 0.00184 | 0.0017 | 0.071 | ADGBto14 | Wean | blautia_producta |
| -0.00657 | 0.00210 | 0.0018 | 0.069 | ADGWto14 | Wean | blautia_producta |
| -0.00284 | 0.00109 | 0.0094 | 0.145 | Week14BF | Wean | blautia_producta |
| -0.72028 | 0.22062 | 0.0011 | 0.053 | Week14Wt | Wean | blautia_producta |
| -0.00010 | 0.00004 | 0.0068 | 0.175 | ADGBto14 | Wean | lactobacillus_reuteri |
| -0.00011 | 0.00004 | 0.0080 | 0.185 | ADGWto14 | Wean | lactobacillus_reuteri |
| -0.01188 | 0.00438 | 0.0068 | 0.159 | Week14Wt | Wean | lactobacillus_reuteri |
| 0.00106 | 0.00042 | 0.0108 | 0.148 | Week14BF | Wean | mitsuokella_multacida |
| 0.00092 | 0.00031 | 0.0035 | 0.118 | ADG14to22 | Wean | trueperella_pyogenes |
| -0.00535 | 0.00170 | 0.0017 | 0.049 | ADGBto14 | 15wk | blautia_producta |
| -0.00618 | 0.00195 | 0.0016 | 0.045 | ADGWto14 | 15wk | blautia_producta |
| -0.64505 | 0.20544 | 0.0017 | 0.044 | Week14Wt | 15wk | blautia_producta |
| -0.00107 | 0.00042 | 0.0104 | 0.198 | Week14BF | 15wk | clostridium_sp_id |
| -0.00210 | 0.00079 | 0.0080 | 0.152 | Week22BF | 15wk | clostridium_sp_id |
| -0.00462 | 0.00154 | 0.0027 | 0.062 | ADGBto14 | 15wk | clostridium_sp_shc |
| -0.00527 | 0.00176 | 0.0029 | 0.065 | ADGWto14 | 15wk | clostridium_sp_shc |
| -0.01711 | 0.00625 | 0.0063 | 0.122 | Week14LEA | 15wk | clostridium_sp_shc |
| -0.57509 | 0.18552 | 0.0020 | 0.045 | Week14Wt | 15wk | clostridium_sp_shc |
| -0.75784 | 0.26076 | 0.0037 | 0.122 | Week22Wt | 15wk | clostridium_sp_shc |
| 0.00307 | 0.00111 | 0.0058 | 0.122 | Week14LEA | 15wk | coprococcus_comes |
| 0.00102 | 0.00034 | 0.0031 | 0.144 | Week22LD | 15wk | coprococcus_comes |
| -0.00113 | 0.00029 | 0.0001 | 0.007 | ADGBto14 | 15wk | eubacterium_coprostanoligenes |
| -0.00130 | 0.00034 | 0.0001 | 0.007 | ADGWto14 | 15wk | eubacterium_coprostanoligenes |
| -0.00058 | 0.00018 | 0.0011 | 0.044 | Week14BF | 15wk | eubacterium_coprostanoligenes |
| -0.00325 | 0.00119 | 0.0064 | 0.122 | Week14LEA | 15wk | eubacterium_coprostanoligenes |
| -0.13155 | 0.03541 | 0.0002 | 0.01 | Week14Wt | 15wk | eubacterium_coprostanoligenes |
| -0.14592 | 0.04977 | 0.0034 | 0.122 | Week22Wt | 15wk | eubacterium_coprostanoligenes |
| -0.00261 | 0.00072 | 0.0003 | 0.013 | ADGBto14 | 15wk | mogibacterium_neglectum |
| -0.00300 | 0.00082 | 0.0003 | 0.013 | ADGWto14 | 15wk | mogibacterium_neglectum |
| -0.00147 | 0.00043 | 0.0007 | 0.038 | Week14BF | 15wk | mogibacterium_neglectum |
| -0.01170 | 0.00292 | 0.0001 | 0.004 | Week14LEA | 15wk | mogibacterium_neglectum |
| -0.32292 | 0.08660 | 0.0002 | 0.01 | Week14Wt | 15wk | mogibacterium_neglectum |
| 0.00612 | 0.00075 | 0.0000 | 0 | ADGBto14 | 15wk | peptococcus_niger |
| 0.00703 | 0.00086 | 0.0000 | 0 | ADGWto14 | 15wk | peptococcus_niger |
| 0.00461 | 0.00045 | 0.0000 | 0 | Week14BF | 15wk | peptococcus_niger |
| 0.01662 | 0.00305 | 0.0000 | 0 | Week14LEA | 15wk | peptococcus_niger |
| 0.73123 | 0.09060 | 0.0000 | 0 | Week14Wt | 15wk | peptococcus_niger |
| 0.00795 | 0.00085 | 0.0000 | 0 | Week22BF | 15wk | peptococcus_niger |
| 0.82077 | 0.12734 | 0.0000 | 0 | Week22Wt | 15wk | peptococcus_niger |
| -0.00188 | 0.00058 | 0.0011 | 0.043 | ADGBto14 | 15wk | prevotella_sp_djf_b |
| -0.00206 | 0.00066 | 0.0020 | 0.05 | ADGWto14 | 15wk | prevotella_sp_djf_b |
| -0.00185 | 0.00067 | 0.0062 | 0.156 | Week14LD | 15wk | prevotella_sp_djf_b |
| -0.00750 | 0.00235 | 0.0014 | 0.055 | Week14LEA | 15wk | prevotella_sp_djf_b |
| -0.22866 | 0.06970 | 0.0011 | 0.041 | Week14Wt | 15wk | prevotella_sp_djf_b |
| 0.05606 | 0.01921 | 0.0036 | 0.073 | ADGBto14 | 15wk | trueperella_pyogenes |
| 0.06313 | 0.02202 | 0.0042 | 0.08 | ADGWto14 | 15wk | trueperella_pyogenes |
| 6.58603 | 2.31837 | 0.0046 | 0.087 | Week14Wt | 15wk | trueperella_pyogenes |
| -0.00890 | 0.00331 | 0.0074 | 0.184 | ADG14to22 | 22wk | blautia_producta |
| -0.00759 | 0.00275 | 0.0058 | 0.13 | ADG14toMKT | 22wk | blautia_producta |
| 0.00717 | 0.00268 | 0.0076 | 0.102 | Week14BF | 22wk | butyrivibrio_crossotus |
| 0.01313 | 0.00472 | 0.0055 | 0.103 | Week22BF | 22wk | butyrivibrio_crossotus |
| -0.00458 | 0.00147 | 0.0019 | 0.063 | ADG14to22 | 22wk | clostridium_sp_id |
| -0.00613 | 0.00272 | 0.0245 | 0.178 | Week14BF | 22wk | clostridium_sp_id |
| -0.00151 | 0.00051 | 0.0030 | 0.057 | Week14BF | 22wk | clostridium_sp_id |
| -0.01158 | 0.00413 | 0.0051 | 0.144 | Week22LEA | 22wk | clostridium_sp_id |
| -0.44813 | 0.13507 | 0.0009 | 0.042 | Week22Wt | 22wk | clostridium_sp_id |
| -0.14464 | 0.04758 | 0.0024 | 0.137 | Week22LD | 22wk | mitsuokella_multacida |
| -0.63260 | 0.19883 | 0.0015 | 0.051 | Week22LEA | 22wk | mitsuokella_multacida |
| 0.00294 | 0.00081 | 0.0003 | 0.012 | ADG14to22 | 22wk | peptococcus_niger |
| 0.00237 | 0.00067 | 0.0005 | 0.015 | ADG14toMKT | 22wk | peptococcus_niger |
| 0.00214 | 0.00048 | 0.0000 | 0.001 | ADGBto14 | 22wk | peptococcus_niger |
| 0.00240 | 0.00055 | 0.0000 | 0.001 | ADGWto14 | 22wk | peptococcus_niger |
| 0.00137 | 0.00028 | 0.0000 | 0 | Week14BF | 22wk | peptococcus_niger |
| 0.25077 | 0.05763 | 0.0000 | 0.001 | Week14Wt | 22wk | peptococcus_niger |
| 0.00346 | 0.00050 | 0.0000 | 0 | Week22BF | 22wk | peptococcus_niger |
| 0.41034 | 0.07454 | 0.0000 | 0 | Week22Wt | 22wk | peptococcus_niger |
| -0.00719 | 0.00213 | 0.0008 | 0.025 | Week22BF | 22wk | prevotella_sp_djf_b |
| -0.00691 | 0.00234 | 0.0033 | 0.148 | Week22LD | 22wk | prevotella_sp_djf_b |
| -0.87940 | 0.32054 | 0.0062 | 0.199 | Week22Wt | 22wk | prevotella_sp_djf_b |

**Supplementary Table 1.** Significant associations for OTUs at different time points at an FDR <0.20 (Benjamini-Hotcheberg). Each OTU trait combination was analyzed with a linear model that included fixed effects of: sex (2 levels); replicate (6 levels); family (28 levels) and the covariate of weight at weaning.


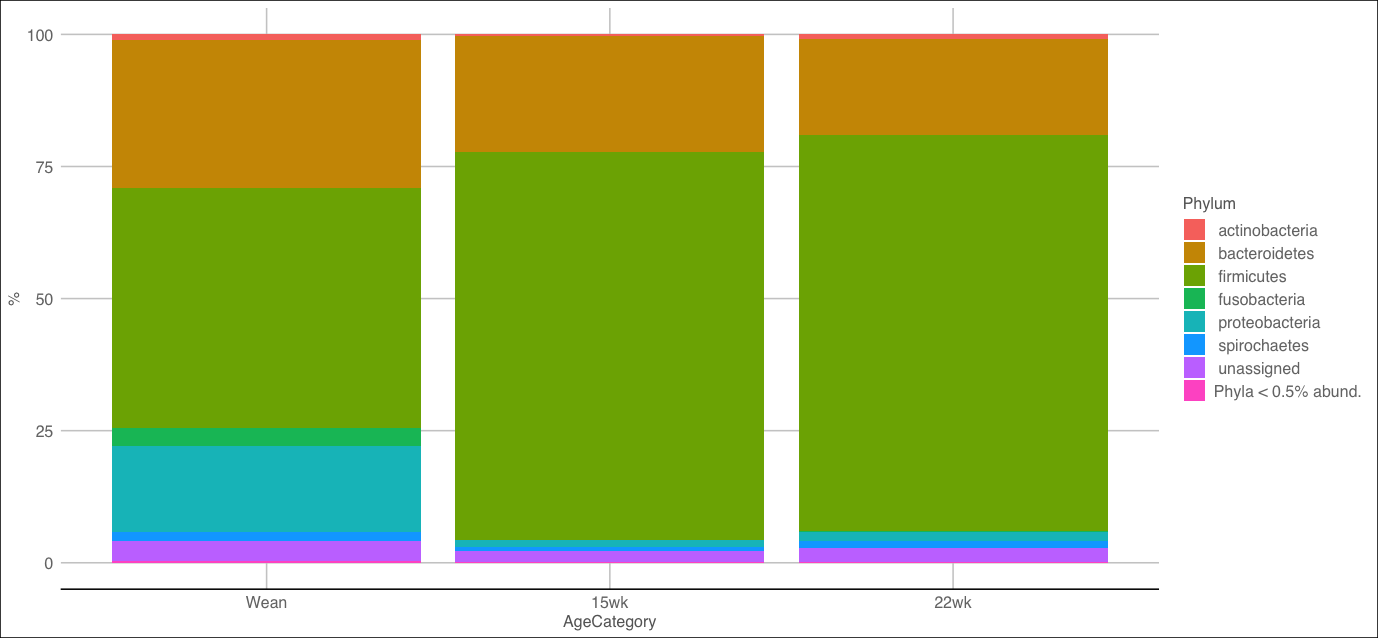


A


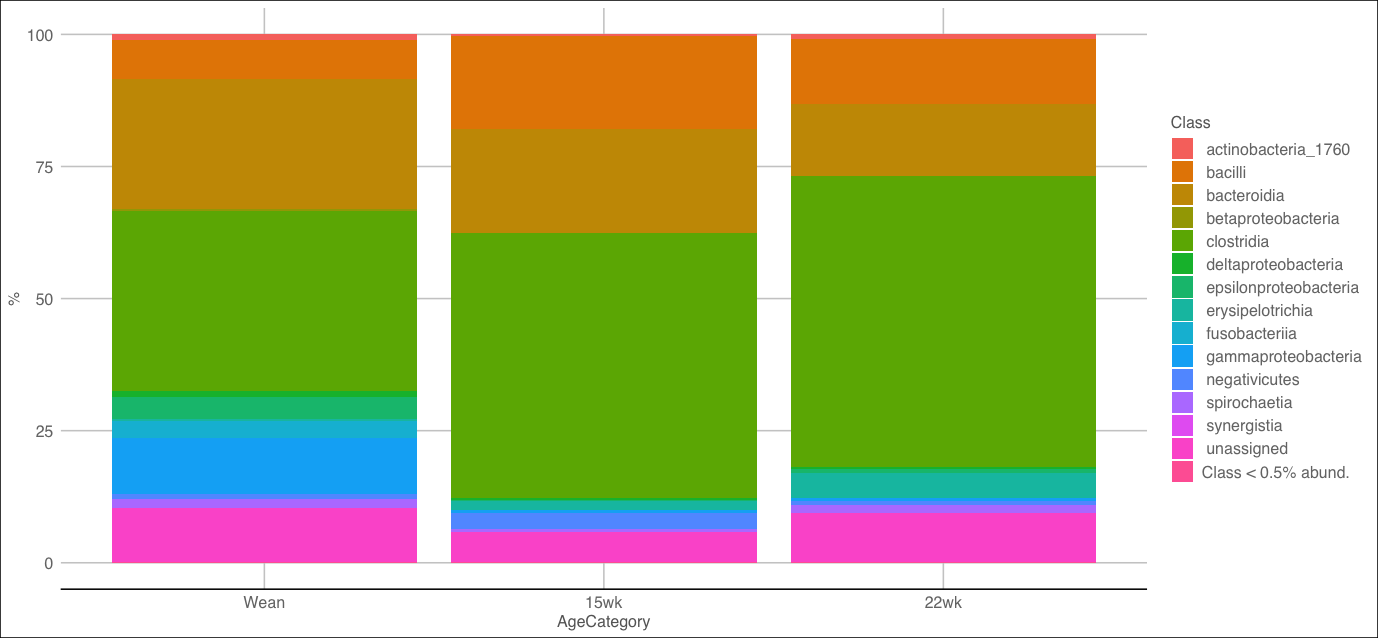


B

**Supplementary Figure 1.**  Taxonomic abundances at different time points (Wean, 15wk, 22wk) at the phylum (Panel A) and class (Panel B) levels.


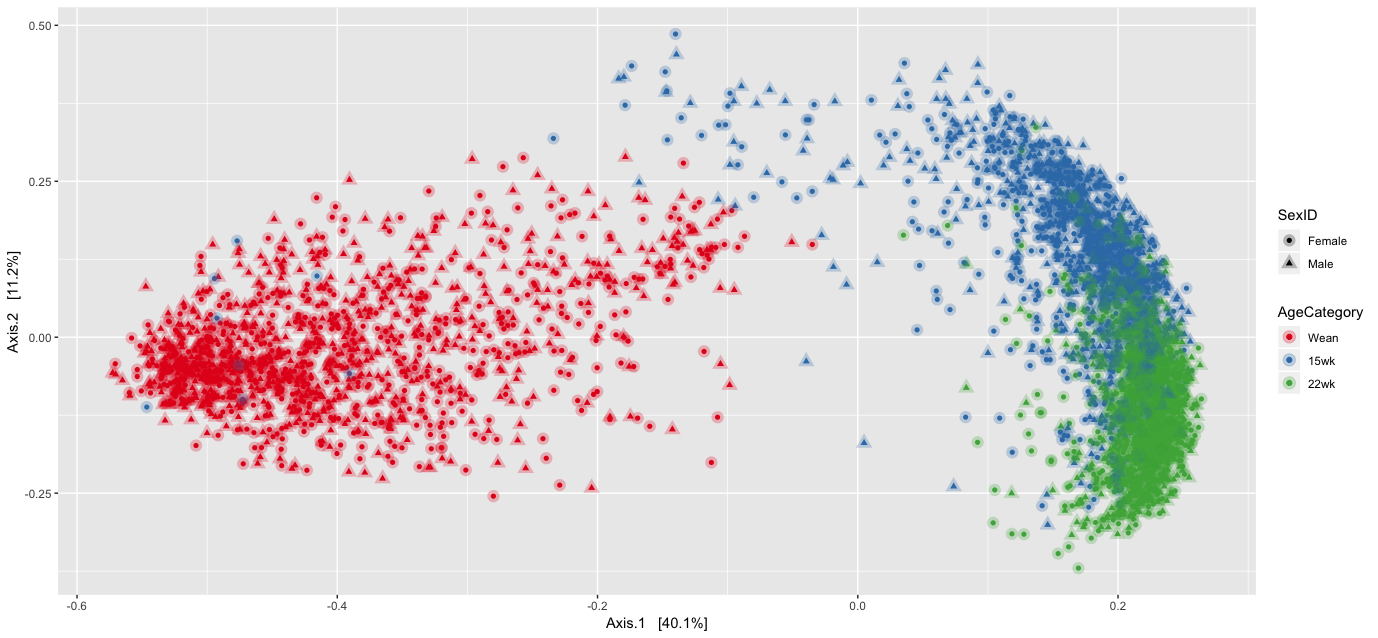


A


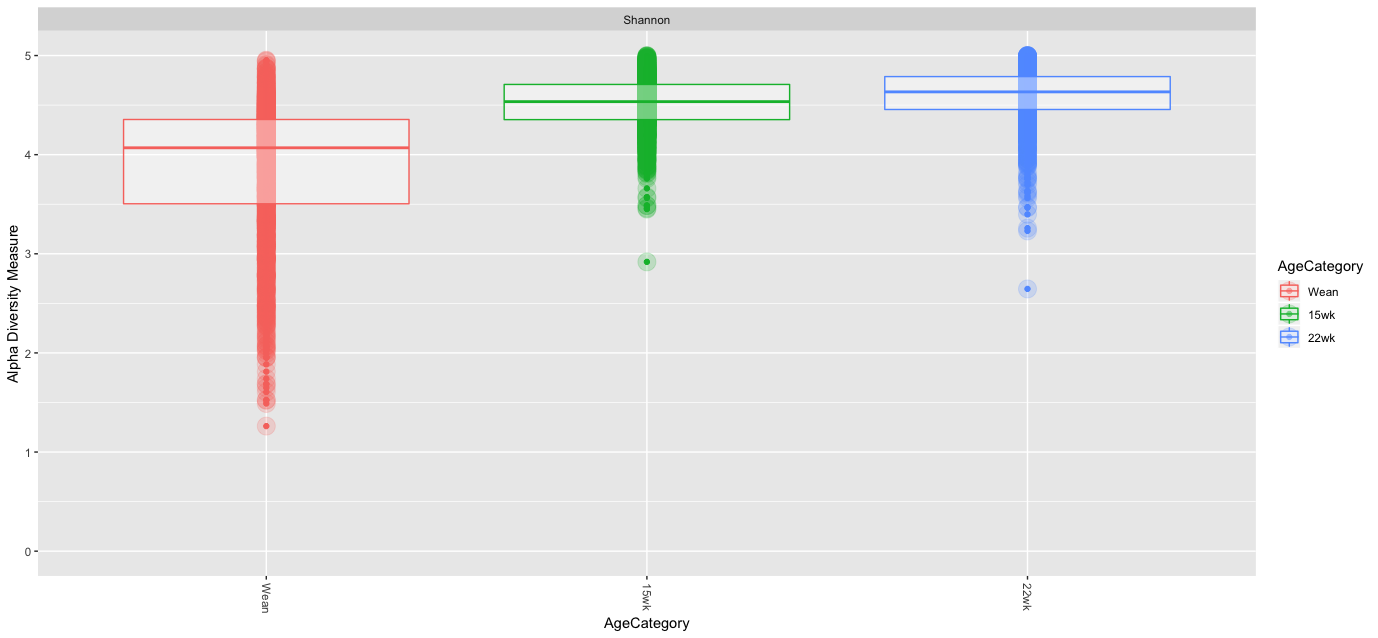


B

**Supplementary Figure 2.**  Principal coordinate analysis illustrating data in terms of age (Wean, 15 weeks, 22 weeks) and sex (male and female) (Panel A). Alpha diversity (Shannon) distributions for each age category in this study (Panel B).


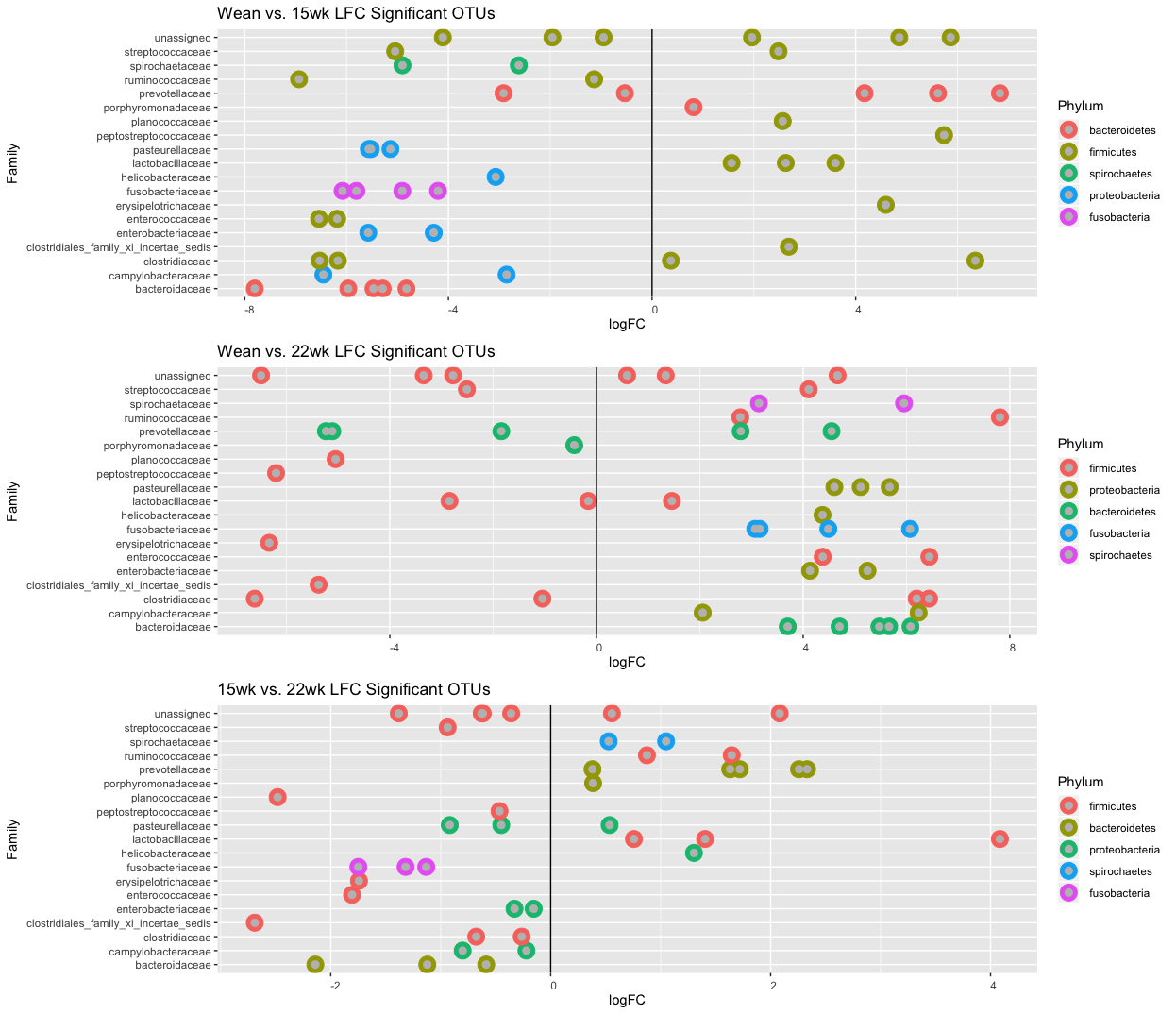


**Supplementary Figure 3.**  The 20 most significant family log fold changes (FDR 0.10) identified when comparing wean vs. week 15 (top panel), wean vs. week 22 (middle panel), and week 15 vs. week 22 (bottom panel).
